# A misdiagnosed case of blastic plasmacytoid dendritic cell neoplasm experiencing multiple recurrences who underwent allogeneic stem cell transplantation: a case report

**DOI:** 10.1186/s13256-021-02860-z

**Published:** 2021-05-23

**Authors:** Fateme Salemi, Seyed Mohammad Reza Mortazavizadeh, Seyyedmohammadsadeq Mirmoeeni, Amirhossein Azari Jafari, Farid Kosari, Seyed Sina Naghibi Irvani

**Affiliations:** 1 Student Research Committee, School of Medicine, Islamic Azad University of Medical Sciences, Yazd, Iran; 2 Department of Hematology and Oncology, Islamic Azad University, Yazd Branch, Yazd, Iran; 3 Student Research Committee, School of Medicine, Shahroud University of Medical Sciences, Shahroud, Iran; 4 Department of Pathology, School of Medicine, Tehran University of Medical Sciences, Tehran, Iran; 5 Research Institute for Endocrine Science, Shahid Beheshti University of Medical Sciences, P.O. Box: 1567812907, Tehran, Iran

**Keywords:** Blastic plasmacytoid dendritic cell neoplasm, Immunohistochemistry, Recurrence, Graft versus host disease

## Abstract

**Background:**

Blastic plasmacytoid dendritic cell neoplasm represents a rare type of hematologic malignancy that often manifests itself through various skin lesions. It commonly affects the elderly male population. Lymph nodes, peripheral blood, and bone marrow involvement are the typical findings that justify its aggressive nature and dismal prognosis. On histopathological assessment, malignant cells share some similarities with blastic cells from the myeloid lineage that make immunohistochemistry staining mandatory for blastic plasmacytoid dendritic cell neoplasm diagnosis.

**Case presentation:**

A 35-year-old Asian man presented with cervical lymphadenopathy followed by an erythematous lesion on his left upper back. At first, the lesion was misdiagnosed as an infectious disease and made the patient receive two ineffective courses of azithromycin and clarithromycin. Six months later, besides persistent skin manifestations, he felt a cervical mass, which was misdiagnosed as follicular center cell lymphoma. Tumor recurrence following the chemoradiation questioned the diagnosis, and further pathologic assessments confirmed blastic plasmacytoid dendritic cell neoplasm. The second recurrence occurred 3 months after chemotherapy. Eventually, he received a bone marrow transplant after complete remission. However, the patient expired 3 months after transplant owing to the third recurrence and gastrointestinal graft versus host disease complications.

**Conclusions:**

Early clinical suspicion and true pathologic diagnosis play a crucial role in patients’ prognosis. Moreover, allogenic bone marrow transplant should be performed with more caution in aggressive forms of blastic plasmacytoid dendritic cell neoplasm because of transplant side effects and high risk of cancer recurrence.

## Background

Comprising only 0.44% of all hematological malignancies, blastic plasmacytoid dendritic cell neoplasm (BPDCN) manifests as a life-threatening form of hematologic malignancy with an insignificant survival rate (usually less than a year). During its aggressive course, BPDCN often presents with multiple erythematous papules and tends to involve peripheral blood, bone marrow, and lymph nodes [1]. In 2008, the World Health Organization changed the tumor’s name from acute myelogenous leukemia to BPDCN and classified it under hematopoietic and lymphoid organ tumors [2, 3]. Since BPDCN demonstrates some overlapping features with other hematologic malignancies, early tumor detection remains challenging. Therefore, a complete history and a physical examination should be accompanied by imaging modalities, flow cytometry, and immunohistochemistry (IHC) of affected tissues to confirm the diagnosis [4–6].

For local and systemic elimination of the neoplastic cells, various therapeutic managements have been approved, including radiotherapy, chemotherapy (similar to acute myeloid leukemia or acute lymphoblastic leukemia chemotherapy drugs), and alloimmune bone marrow stem cell transplantation [7–9]. Here, we present a rare and sophisticated case of a patient with misdiagnosed BPDCN, who experienced multiple recurrences during treatment.

## Case presentation

**Fig. 1:**
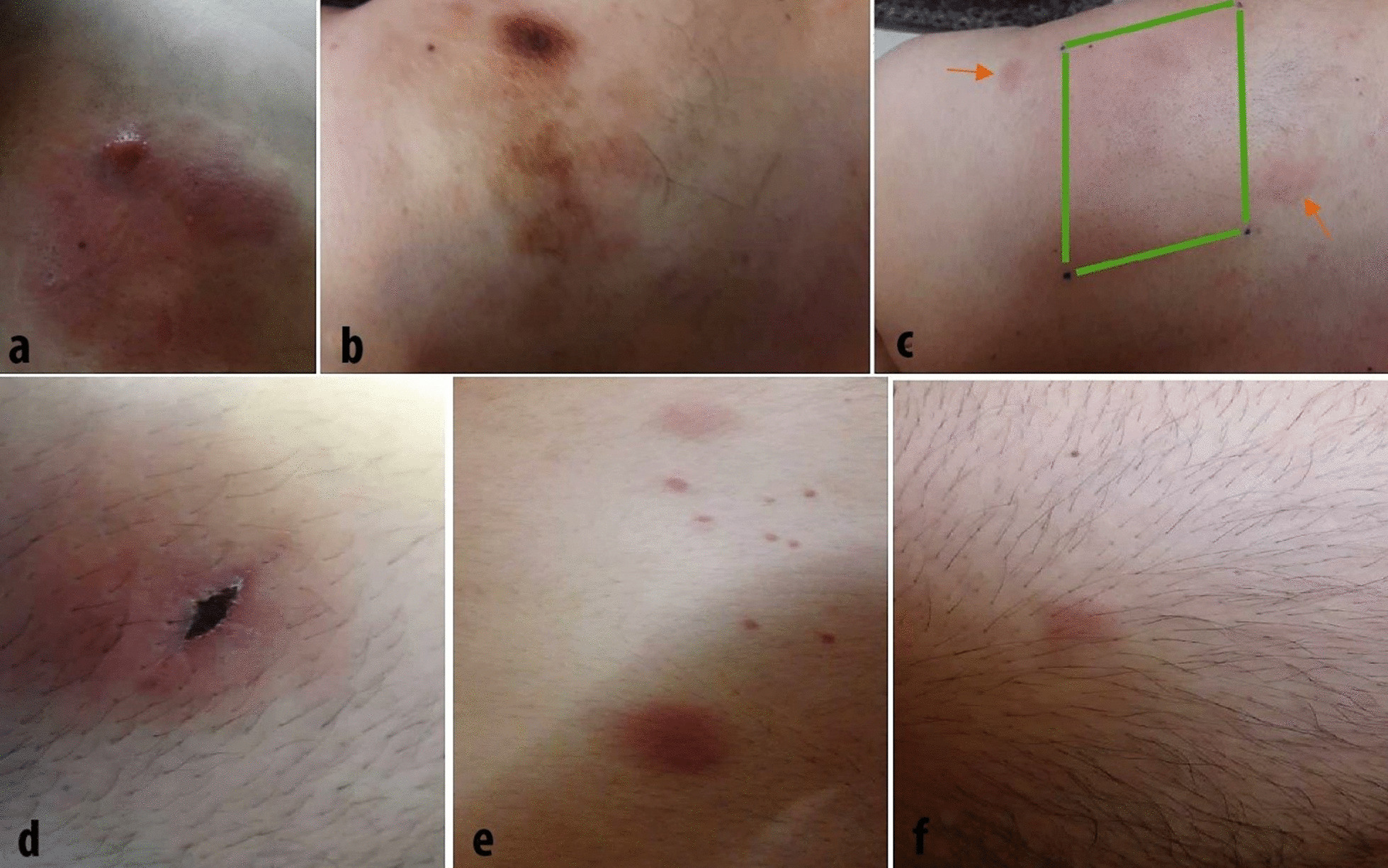
Infiltrative erythematous plaque and a nodule on the left upper shoulder (**a**), which changes color over time (**b**); ill-defined erythematous nodules (arrows) out of the radiotherapy zone (square) (**c**); ill-defined erythematous nodule on the dorsal aspect of left leg (the crust is from skin biopsy) (**d**); multiple erythematous nodules on the back (**e**) and right thigh (**f**)

In May 2017, a 35-year-old Asian man presented with an infiltrative erythematous plaque and a nodule on his left upper shoulder. He had no fever, chills, or any other symptoms (Fig. 1a, b). At first, he received two courses of azithromycin (Azithrocin) and clarithromycin (Biaxin) (500 mg orally on day 1 followed by 250 mg orally daily on days 2–5 for both antibiotics) to rule out possible infection. During 6 months, besides the lack of response, a palpable cervical mass appeared that was persistent to antimicrobial treatment. Therefore, 2 months later, he was referred to an oncologist for further evaluation. Despite insignificant past medical and family history, complete blood count, and peripheral blood smear, he showed cervical and supraclavicular lymphadenopathy on physical examination.

Cervical lymph node biopsy diagnosis showed diffuse malignant lymphoma, and IHC staining confirmed the tumor subtype as follicular center cell lymphoma (a non-Hodgkin lymphoma).On IHC, malignant cells displayed positive immunoreactivity for CD10, CD20, BCL2, TdT, LCA, and ki67 and negative immunoreactivity for CD3, CD4, CD5, CD16, CD30, CD56, CD79a, BCL6, and PAX-5 (Table 1).

**Table 1:** Histopathological and IHC findings of disease from onset to last recurrence

	Disease onset	First relapse	Second relapse	Third relapse
Skin involvement	Infiltrative erythematous plaque and nodule on left upper shoulder	Ill-defined erythematous nodules on left upper shoulder outside the radiotherapy margin	Two new erythematous nodules on dorsal aspects of left forearm and left leg	Multiple erythematous nodules on upper back, dorsal aspect of right thigh and ventral aspect of left leg
Other clinical symptoms	Cervical lymphadenopathy	–	–	–
Histopathology	Lymph node biopsy: not mentioned	Skin biopsy: focal intradermal monomorphous infiltrate of medium-sized blast-like cells without any significant mitotic activity or necrosis	Like the first relapse	Like the first relapse
Immunohistochemistry	Positive: CD10, CD20, BCL2, TdT, LCA, ki67	Positive: CD43, CD4, ki67, CD68, CD33, CD56, CD123	Like the first relapse	Like the first relapse
	Negative: CD3, CD4, CD5, CD16, CD30, CD56, CD79a, BCL6 , PAX-5	Negative: CD20, CD3, CD30, CD5, CD34, PAX5, CD79a, MPO, Pan CK, Melan A, CD56, TdT, C-kit, CD138, PAX5, CD79a, LCA	–	–

Analysis of bone marrow aspiration and biopsy confirmed the lack of any infiltrative lesion or malignancy. Nevertheless, thoracic and abdominopelvic computed tomography (CT) scans illustrated an enlargement in the left side of the neck and supraclavicular lymph nodes (up to 24 mm in diameter), a nasopharyngeal soft tissue thickening, and a hypodense right liver lobe lesion.

The patient received eight courses of rituximab, cyclophosphamide, doxorubicin, vincristine, and prednisone (R-CHOP) chemotherapy regimen and radiotherapy (3600 cGy in 18 fractures) with 6 MeV electron beams. After treatment, skin lesions as well as lymphadenopathy and subcutaneous chest wall infiltrations disappeared. However, small liver lesions did not disappear in the follow-up CT scan. The absence of pathologic hypermetabolic lesions on positron emission tomography–CT (PET-CT) scan was in favor of remission. However, 3 months later, two ill-defined erythematous nodules appeared out of the radiotherapy zone (Fig. 1c), and their histopathology was compatible with BPDCN.

**Fig. 2:**
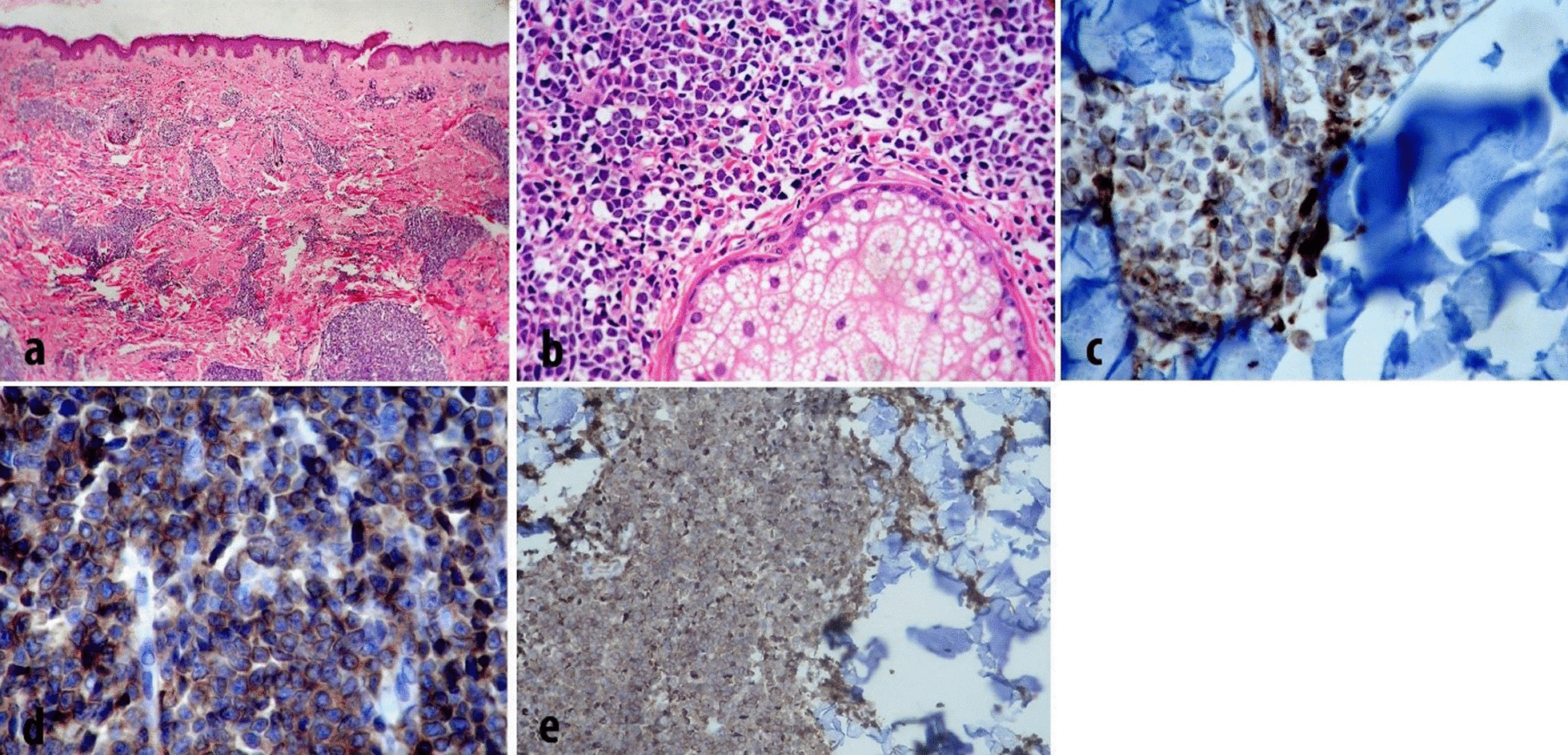
Skin biopsy of left forearm showing dense dermal periadnexal infiltration of blastoid tumor cells (**a**), with medium-sized vesicular nuclei, smooth chromatin and scanty cytoplasm (**b**). The tumor cells show positive CD68 (para nuclear dot-like pattern) (**c**), CD4 (**d**), and CD123 (**e**)

To clarify the controversy in diagnosis, the third pathologist reviewed the first and second specimens and confirmed BPDCN as the true diagnosis. Skin biopsy showed that medium-sized monomorphic blast-like cells focally infiltrated the dermis without any significant signs of mitotic activity or necrosis (Fig. 2a, b). IHC staining of the lesion revealed diffuse positive immunostaining of atypical cells with CD43 and CD4. The tumor also showed scattered ki67 staining (5–10%) and focal dot-like cytoplasmic forms in CD68 immunostaining with weakly positive CD33, CD56, CD123, and LCA. These CD markers were absent: CD20, CD3, CD30, CD5, CD34, PAX5, CD79a, MPO, Pan CK, Melan A, TdT, C-kit, and CD138. Negative LCA and B-cell markers including PAX5, CD20, and CD79a in the IHC study panel made any kind of B-cell lymphoma unlikely (Fig. 2c–e). Therefore, two courses of hyper-CVAD (cyclophosphamide, vincristine, adriamycin, dexamethasone) started to make the patient an eligible candidate for allogeneic stem cell transplantation (SCT) after complete remission.

During treatment, the patient showed signs of remission: normal peripheral blood smear and flow cytometry of bone marrow cells, severely hypocellular marrow in bone marrow aspiration and biopsy, and acellular cerebrospinal fluid (CSF). However, grade 1 fatty liver and elevated serum transaminases postponed the transplant. Blood culture following febrile neutropenia found *Klebsiella pneumoniae*; thus, the patient received daily ciprofloxacin 500 mg orally during that period. Meanwhile, the patient found two new erythematous nodules on the dorsal aspect of his left forearm and left leg, which was confirmed as the second recurrence in biopsy (Fig. 1d). Despite the indication of target therapy with daratumumab and tagraxofusp, the patient received three courses of ifosfamide, carboplatin, and etoposide (ICE)chemotherapy owing to financial issues.

Consequently, following complete remission and normal liver transaminase levels, he underwent SCT from a full-match donor (his sister) as the first eligible case of BPDCN for transplant in Iran. Unfortunately, 2 months later, new skin lesions appeared that made graft versus host disease (GVHD) a probable etiology. However, biopsy confirmed the third recurrence of malignant cells (Fig. 1e, f), and the patient restarted the treatment. A few months later, he experienced severe watery diarrhea and underwent a diagnostic workup for gastrointestinal GVHD. Stool culture ruled out bacterial, fungal, and viral pathogens, and colonoscopy displayed erosion and necrosis of bowel mucosa with shallow ulcers. After the confirmed diagnosis of GVHD, chemotherapy was stopped and the patient received GVHD treatment according to protocols of the American Society of Hematology [10]. Unfortunately, 3 months later, the patient died from GVHD and tumor recurrence complications.

## Discussion and conclusions

We have reported a case of BPDCN in a 37-year-old man who presented with a cutaneous lesion at disease onset. BPDCN is an unusual hematologic neoplasm that usually involves patients older than 60 years and has male predominance. Hence, it has a lower incidence among children and adults younger than 50 years old. This tumor initially involves the skin with a nonspecific morphology and distribution ranging from erythematous, purplish papules to bruise-like plaques or patches with size variability (from a few millimeters to several centimeters) and no inclination toward any specific anatomical area [11].

Unusual cutaneous and extracutaneous manifestations have been reported in some cases, including gingival lesions, acute rheumatic fever, pulmonary, central nervous system (CNS), and testicular infiltration [12, 13]. Besides, neoplastic cells metastasize to peripheral blood and bone marrow, lymph nodes, and spleen in 60–90%, 56%, and 34–78% of cases, respectively. Bone marrow is spared at disease onset, though it might be involved as the cancer progresses towards the leukemic phase, which indicates a poor prognosis [14]. Yet, our patient’s bone marrow was clear of neoplastic cells and became severely hypocellular as a result of cytotoxic chemotherapy. In BPDCN, peripheral blood may contain medium-sized blasts that have scant cytoplasm, fine nuclear chromatin, and evident nucleoli [15]. On flow cytometry, most of the neoplastic cells that overexpress CD123 may express a positive reactivity for CD4, CD56, CD303, or TCL1. The neoplastic cells derive from rapidly expanding progenitors of plasmacytoid dendritic cells that originate from the myeloid lineage [16].

Although histopathology showing lymphoblasts or small immunoblots supports the presence of a neoplastic reaction, it requires IHC staining to verify the diagnosis and distinguish this malignancy from its differential diagnoses [17, 18]. The differential diagnoses of BPDCN include mature T-cell lymphoma, myeloid sarcoma, acute myeloid leukemia, and T-cell lymphoblastic lymphoma [19]. Unlike BPDCN, T-cell biomarkers, including CD3, CD1a, and CD10, are detected in T-cell lymphoblastic lymphoma. Nevertheless, instead of skin involvement, T-cell lymphoblastic lymphoma often presents with a mediastinal mass. As an extramedullary myeloid tumor, myeloid sarcoma consists of myeloblasts or premature myeloid cells [20]. Monoblastic lineage of both BPDCN and myeloid sarcoma express CD56, CD123, and CD4 markers; nevertheless, TdT is exclusively detected in 33% of the BPDCN patients. Despite IHC, clinical manifestations can distinguish myeloid sarcoma from BPDCN. Unlike lymphoid leukemia, myeloid leukemia neoplastic cells, especially the monoid lineage L4 and L5 may share some features in common with blastic plasmacytoid dendritic cells. However, monoblastic leukemia (AML-M5) originates from bone marrow and may metastasize to the skin. On the other hand, BPCDN has a dermal origin and may infiltrate into bone marrow as the disease evolves [19].

Currently, due to insufficient existing data regarding BPDCN and lack of treatment protocols, the patient underwent conventional polychemotherapy (acute lymphoblastic leukemia (ALL)-type, acute myeloid leukemia (AML)-type, and lymphoma-type therapy). In terms of survival outcomes and complete remission rates, ALL treatment is preferred to AML-type treatment. Complete remission rates vary from 55% to 80% in CHOP or CHOP-like regimens; however, recurrence-free survival rarely lasts more than 5 months [7, 21]. In 2018, the FDA approved tagraxofusp or SL401 as the standard treatment for BPDCN since it targets CD123-expressing cells [22]. *In vivo* and *in vitro* studies had promising results regarding the efficacy of this drug from phase I and II clinical trials, showing significant improvement in overall survival compared with conventional BPDCN chemotherapy regimens. Tagraxofusp also has some notable side effects, including hypoalbuminemia, elevated transaminases, thrombocytopenia, and capillary leak syndrome [23–25]. Allogeneic or autologous transplant after first complete remission leads to long-term overall survival; nevertheless, from a practical point of view, this may not be feasible owing to advanced age at presentation [26]. Radiotherapy locally eradicates intradermal infiltration, which has shown promising consequences in patients with mere cutaneous manifestations [27].

Despite various therapeutic options, a high recurrence rate has caused a dismal prognosis for BPDCN, which is due to the tumor’s unspecific cutaneous presentation, aggressive nature, and challenging diagnosis in most patients. Unfortunately, in this case, besides antibiotic resistance, unjustified antimicrobial treatment provoked rapid tumor expansion and multiple recurrences despite high-dose chemo- and radiotherapy. In addition, allogeneic bone marrow SCT has promising results in patients without recurrence. Nevertheless, its disadvantages outweigh the advantages in recurrent BPDCN, since it imposes likely tumor relapse, GVHD complications, and systemic infections on these patients.

In conclusion, this case highlights the significance of early diagnosis and treatment in BPDCN outcome. Multiple recurrences adversely affect patients’ prognosis; therefore, bone marrow transplant options should be reserved for those without recurrences. In relapsing and aggressive forms, transplant side effects as well as tumor recurrence should be considered to prevent further complications and extra costs.

## Data Availability

The case report data is not publicly available, but it could be available from the corresponding author upon reasonable request.

## Abbreviations

BPDCN: Blastic plasmacytoid dendritic cell neoplasm
IHC: Immunohistochemistry
PET-CT: Positron emission tomography–computed tomography
SCT: Stem cell transplantation
CSF: Cerebrospinal fluid
CBC: Cell blood count
GVHD: Graft versus host disease
CNS: Central nervous system
AML: Acute myeloid leukemia
ALL: Acute lymphoblastic leukemia
CT: Computed tomography
